# Photoexcitation-induced passivation of SnO_2_ thin film for efficient perovskite solar cells

**DOI:** 10.1093/nsr/nwad245

**Published:** 2023-09-13

**Authors:** Nianyao Chai, Xiangyu Chen, Zhongle Zeng, Ruohan Yu, Yunfan Yue, Bo Mai, Jinsong Wu, Liqiang Mai, Yi-Bing Cheng, Xuewen Wang

**Affiliations:** State Key Laboratory of Advanced Technology for Materials Synthesis and Processing, International School of Materials Science and Engineering, Wuhan University of Technology, Wuhan430070, China; State Key Laboratory of Advanced Technology for Materials Synthesis and Processing, International School of Materials Science and Engineering, Wuhan University of Technology, Wuhan430070, China; State Key Laboratory of Advanced Technology for Materials Synthesis and Processing, International School of Materials Science and Engineering, Wuhan University of Technology, Wuhan430070, China; State Key Laboratory of Advanced Technology for Materials Synthesis and Processing, International School of Materials Science and Engineering, Wuhan University of Technology, Wuhan430070, China; State Key Laboratory of Advanced Technology for Materials Synthesis and Processing, International School of Materials Science and Engineering, Wuhan University of Technology, Wuhan430070, China; State Key Laboratory of Advanced Technology for Materials Synthesis and Processing, International School of Materials Science and Engineering, Wuhan University of Technology, Wuhan430070, China; State Key Laboratory of Advanced Technology for Materials Synthesis and Processing, International School of Materials Science and Engineering, Wuhan University of Technology, Wuhan430070, China; State Key Laboratory of Advanced Technology for Materials Synthesis and Processing, International School of Materials Science and Engineering, Wuhan University of Technology, Wuhan430070, China; National Energy Key Laboratory for New Hydrogen-Ammonia Energy Technologies, Foshan Xianhu Laboratory, Foshan528000, China; State Key Laboratory of Advanced Technology for Materials Synthesis and Processing, International School of Materials Science and Engineering, Wuhan University of Technology, Wuhan430070, China; National Energy Key Laboratory for New Hydrogen-Ammonia Energy Technologies, Foshan Xianhu Laboratory, Foshan528000, China; State Key Laboratory of Advanced Technology for Materials Synthesis and Processing, International School of Materials Science and Engineering, Wuhan University of Technology, Wuhan430070, China; National Energy Key Laboratory for New Hydrogen-Ammonia Energy Technologies, Foshan Xianhu Laboratory, Foshan528000, China

**Keywords:** photoexcitation-induced passivation, ultrafast laser, carrier management, electron transport layer, perovskite solar cells

## Abstract

A high-quality tin oxide electron transport layer (ETL) is a key common factor to achieve high-performance perovskite solar cells (PSCs). However, the conventional annealing technique to prepare high-quality ETLs by continuous heating under near-equilibrium conditions requires high temperatures and a long fabrication time. Alternatively, we present a non-equilibrium, photoexcitation-induced passivation technique that uses multiple ultrashort laser pulses. The ultrafast photoexcitation and following electron–electron and electron–phonon scattering processes induce ultrafast annealing to efficiently passivate surface and bulk defects, and improve the crystallinity of SnO_2_, resulting in suppressing the carrier recombination and facilitating the charge transport between the ETL and perovskite interface. By rapidly scanning the laser beam, the annealing time is reduced to several minutes, which is much more efficient compared with conventional thermal annealing. To demonstrate the university and scalability of this technique, typical antisolvent and antisolvent-free processed hybrid organic–inorganic metal halide PSCs have been fabricated and achieved the power conversion efficiency (PCE) of 24.14% and 22.75% respectively, and a 12-square-centimeter module antisolvent-free processed perovskite solar module achieves a PCE of 20.26%, with significantly enhanced performance both in PCE and stability. This study establishes a new approach towards the commercialization of efficient low-temperature manufacturing of PSCs.

## Teaser

A new ultrafast annealing strategy to replace high-temperature thermal heating is developed to process SnO2 semiconductor film by femtosecond laser, facilitating the commercial production of efficient perovskite solar cells and other flexible electronics.

## INTRODUCTION

Inorganic metal oxide semiconductors are widely used as electron transport layers (ETLs) for perovskite solar cells (PSCs), which efficiently extract and transport electrons from the perovskite layer into the electrode [1–5]. Tin (IV) oxide (SnO_2_)-based ETLs offer desirable band alignment and electron mobility, while being processable at much lower temperatures [6,7]. Among various deposition methods [8–10], SnO_2_ nanoparticle-based PSCs prepared by using chemical bath deposition (CBD), which provides conformal coverage between the perovskite layer and the electrode, have demonstrated the best performance so far [11–13]. However, the intrinsic defects (e.g. oxygen vacancies, tin interstitials) and surface defects (e.g. hydroxyl groups and Sn dangling bonds) that are unavoidably formed in SnO_2_ generate massive shallow trap states near the conduction band and result in carrier recombination at the SnO_2_/perovskite interface [14,15]. To reduce the carrier loss, the ETL film prepared by using CBD generally requires an annealing condition with a temperature of ≥170°C for ≥1 hour [11–13,16]. This is in part because the ETLs normally show poor heat transfer and large thermal inertia for passivation under near thermal equilibrium, which makes it challenging to reduce the annealing temperature and time, bringing additional energy costs. Currently, except for thermal annealing at high temperatures for at least 1 h, there is no better way to reduce the annealing temperature of tin oxide films prepared by using the CBD method [17,18]. At the same time, thermal annealing is generally followed by buried interface passivation to further reduce surface defects, which requires additional thermal annealing and complicates the preparation process [19,20].

Herein, we report a novel non-equilibrium, photoexcitation-induced passivation (PiP) technique that uses ultrashort laser pulses. The ultrafast photoexcitation and following electron–electron and electron–phonon scattering processes induce an ultrafast electron and phonon heating process, thus ensuring efficient low-temperature annealing of SnO_2_ nanoparticle-based ETLs prepared by using CBD. By rapidly scanning a laser beam, the annealing process of a 5 cm × 5 cm sample can be finished within 30 s at room temperature, which facilitates the scale-up and energy saving of fabricating PSCs. The two representative perovskite-based PSCs fabricated via this method achieve a great enhancement in comparison with the control devices, and a power conversion efficiency (PCE) of 20.26% for a 12-square-centimeter perovskite solar module (PSM) is achieved as well.

## RESULT AND DISCUSSION

### PiP of SnO_2_ thin film

**Figure 1.**
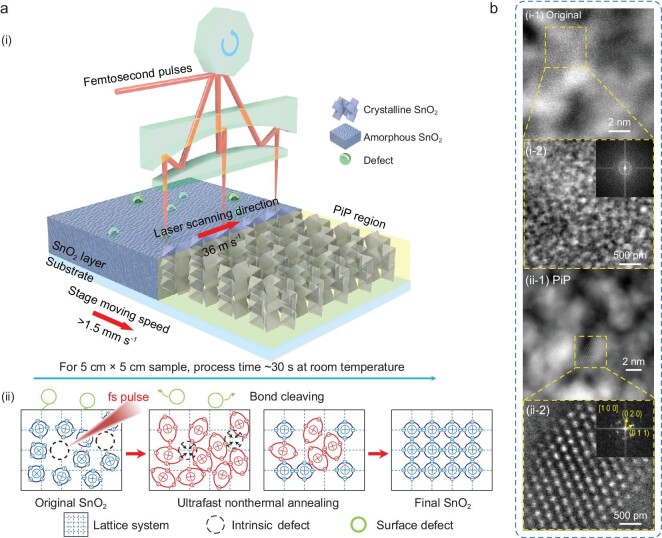
Diagram of the photoexcitation-induced ultrafast passivation (PiP) process and phase transition of SnO_2_ thin films. (a) Schematic of the PiP procedure during the annealing of SnO_2_ thin films and the specific mechanisms of the annealing process including photoexcitation, electron–electron and electron–phonon scattering, rearrangement of atoms and defects passivation. (b) High-resolution TEM images of (i) original-SnO_2_ and (ii) PiP-SnO_2_ (the insets show fast Fourier transform patterns of the TEM images).

The schematic of a home-built PiP set-up and the mechanism of PiP are shown in Fig. 1a. A femtosecond laser beam is reflected by a high-speed rotating polygon mirror and then focused on the sample. The set-up has a laser scanning speed of >36 m s^−1^ in a working range of 300 mm, with a stage movement speed of >1.5 mm s^−1^, which ensures annealing a 5 cm × 5 cm sample in 30 s and a 10 cm × 10 cm sample in 60 s, as shown in Fig. 1a (i). The detailed home-built PiP experimental set-up can be found in the Methods and Supplementary material. The demonstration and actual processing video can be found in the Supplementary movie.

The PiP process is achieved by using ultrafast intensive excitation after laser pulse energy deposition in tens of femtoseconds, followed by the electron–electron and electron–phonon scattering processes (in hundreds of femtoseconds), as shown in Fig. 1a (ii). The interaction between the ultrafast laser and the SnO_2_ film starts from direct photon absorption by excitation of intrinsic free carriers and shallow trap states. The absorption coefficient is 1650 cm^−1^ at 1030 nm as measured. The carrier population will rapidly rise up to the anti-bonding states (conduction band) by impact ionization via the electron–electron scattering process in tens of femtoseconds. The population of anti-bonding states raises the free energy of the excited system, generating a stretching force on each bond, which efficiently cleaves weak or metastable bonds, thus passivating the surface defects and oxygen vacancies of the SnO_2_ film [21–23].

The following electron–phonon scattering process to reach an equilibrium temperature of the electron and phonon system induces an ultrafast phonon heating process (for 28 mJ cm^−2^ laser fluence, the maximum temperature rise is 22.6 K) with a heating rate of >10^13^ K s^−1^ in hundreds of femtoseconds (see the Supplementary material for theoretical analysis of temperature rise by PiP) and an ultrafast thermal gradient with the Gaussian distribution is generated in the focus zone (Supplementary Fig. S1a) [24]. On the way to cooling the excited system, atoms obtain excessive kinetic energy to align into an ordered arrangement, thus passivating the bulk defects of the SnO_2_ and improving the crystallinity of the SnO_2_ [21,25]. This temperature gradient decays rapidly via thermal diffusion in 25 ns with a quenching rate of ∼10^9^ K s^−1^. The accumulation temperature after 50 consecutive laser pulses is only 6.5 K (Supplementary Fig. S1b), showing that the temperature rise after each single pulse drops to near room temperature before the next pulse arrives, demonstrating that the accumulation effect is negligible in the PiP process of the SnO_2_ film. This shows that the whole passivation process is completed very fast with much more energy and time efficiency compared with the conventional annealing technique.

The microscopic characterization of the SnO_2_ thin film cross sections using high-resolution transmission electron microscope (HRTEM) and the corresponding energy-disperse X-ray spectroscopy of Sn, O, F and Pt (Supplementary Fig. S2) present a complete coverage over the fluorine-doped tin oxide (FTO) surface. By comparing the HRTEM images before and after the PiP, it is found that a large number of amorphous particles existed in the original SnO_2_ (Fig. 1b, part i-1). This is more clearly identified from the random distribution of Sn atoms in the atomic-resolution image and the corresponding fast Fourier transform (FFT) pattern (Fig. 1b, part i-2). More representative HRTEM images of the original SnO_2_ are shown in Supplementary Fig. S3. As shown in Fig. 1b, part ii-1, the PiP process leads to the SnO_2_ undergoing a transformation from the amorphous phase to the crystalline phase, thus improving the crystallinity of the SnO_2_, which is confirmed by the massive appearance of lattice fringes and regularly arranged Sn atoms. The FFT pattern of the SnO_2_ atomic-resolution image (Fig. 1b, part ii-2) after PiP corresponds to the [100] zone axes. More representative HRTEM images of the SnO_2_ after the PiP are shown in Supplementary Fig. S4.

### Morphology, optoelectronic properties and surface defect characterization of SnO_2_ films

**Figure 2.**
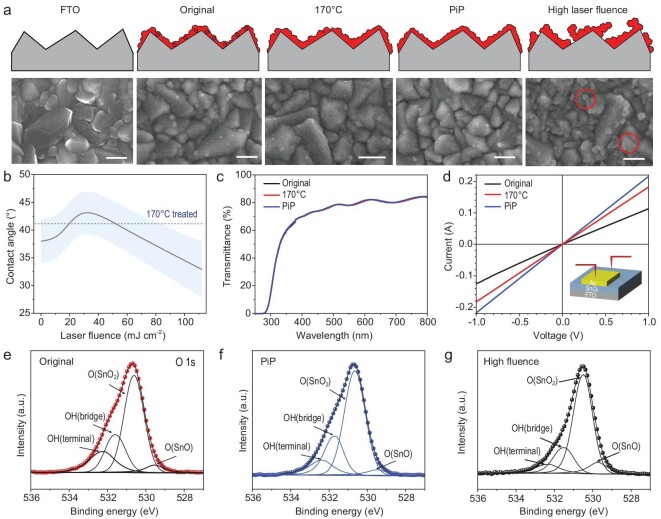
Characterization of SnO_2_ films. (a) Schematic illustration of morphologic change of original-SnO_2_, 170°C-SnO_2_ and PiP-SnO_2_ thin films on FTO substrate and corresponding top-view SEM. Scale bars are 200 nm. Comparison of (b) contact angle, (c) transmittance spectra and (d) conductivity of the original-SnO_2_, 170°C-SnO_2_ and PiP-SnO_2_ thin films on the FTO substrate. (e–g) The XPS spectrum of O1s of original-SnO_2_, PiP-SnO_2_ and high-laser-fluence-treated SnO_2_ thin films.

To investigate the reasons why the PiP-SnO_2_ thin film efficiently improves the cell performance, the surface morphology of SnO_2_ thin films was studied by using scanning electron microscopy (SEM) images, as shown in Fig. 2a. Compared with the smooth, bare FTO surface, the SnO_2_ thin film deposited on the FTO is composed of compact nanoparticles. All SnO_2_ thin films are uniform and full-coverage except for the much higher laser fluence. This is because at high laser fluence, SnO_2_ nanoparticles tend to coalesce into large crystallites, resulting in pinholes in the SnO_2_ thin films. These large crystallites and pinholes act as carrier trap sites in the SnO_2_/perovskite interface where the energy is lost through non-radiative recombination pathways, resulting in a reduction in carrier lifetimes. The contact angle first increases with increasing laser fluence (Fig. 2b), which indicates a reduction in surface hydrophilic groups. However, when the laser fluence is further increased, the non-uniformity of the surface increases the hydrophilicity. The transmittance spectra of the original-SnO_2_, 170°C thermal-annealed SnO_2_ for 1 hour (170°C-SnO_2_) and PiP-SnO_2_ thin films on FTO substrates are shown in Fig. 2c and Supplementary Fig. S5a. There is a slight increase in the transmittance of the substrate with the PiP-SnO_2_ thin films, which could contribute to the high J_SC_ of the PSCs. Since the SnO_2_ thin film is very thin relative to the FTO layer, there is no clear difference in transmittance. The conductivity of the SnO_2_ thin films on the FTO shows an increase of >20% by the PiP (Fig. 2d and Supplementary Fig. S5b). Moreover, the electron mobility of various ETLs measured by using a space charge-limited current method is shown in Supplementary Fig. S6 [26]. It can be found that the electron mobility of the SnO_2_ is higher than that of the original-SnO_2_ and 170°C-SnO_2_, which is beneficial to SnO_2_ thin films to transport electrons generated from the perovskite layer, thereby improving the cell performance.

Since the film deposition takes place in a water-based solution, OH groups (surface defects) inevitably appear in the SnO_2_ thin film, which is proved by the X-ray photoelectron spectrum (XPS) in Fig. 2e–g and Supplementary Fig. S7a. After the calibration of the spectra by using the C1s peak, the deconvolution of the O1s peak was executed in detail to detect the four oxygen species for each sample. Generally, there are two kinds of OH groups commonly existing on the SnO_2_ thin film surface [27]. One is OH_(bridge)_ integrated with two metal sites at 531.5 (±0.1) eV and the other is OH_(terminal)_, which binds to one metal site at 532.2 (±0.1) eV [27–29]. The relative proportions of OH_(bridge)_ and OH_(terminal)_ species for each sample are listed in Supplementary Table S1. The relative proportions of OH_(bridge)_ and OH_(terminal)_ decrease both for 170°C-SnO_2_ and PiP-SnO_2_ thin films, which indicates that the PiP achieves a similar effect to conventional high-temperature thermal annealing. Compared with the 170°C annealing for 1 hour, the PiP is much more time-efficient. From the XPS results, we can confirm that the PiP process can not only drive the SnO_2_ undergoing a phase transition from the amorphous state to the crystalline state, but also reduce the metastable surface functional groups on SnO_2_ thin films, as shown in Supplementary Fig. S7b. The metastable OH_(bridge)_ and OH_(terminal)_ could be cleaving after photoexcitation, which is also indicated by the enhanced Raman peak at 632 cm^−1^ (corresponding to the fundamental active Raman vibration modes A_1g_) in Supplementary Fig. S8 [30,31].

### Carrier transport behaviors between SnO_2_ films and perovskite films

**Figure 3.**
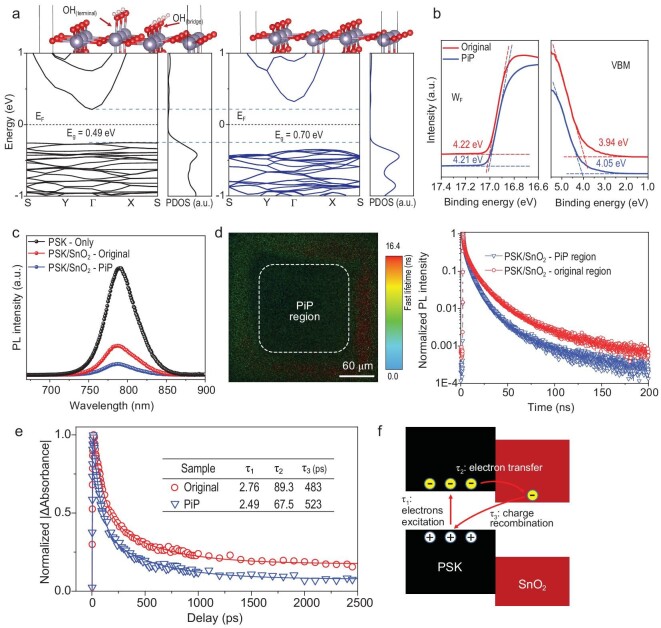
Influence of PiP of SnO_2_ on its band alignment with perovskite and the carrier transfer processes at the interface of the SnO_2_ and the perovskite. (a) Band structure and partial density of states of original-SnO_2_ and PiP-SnO_2_. (b) The energy onset and cut-off of UPS spectra of original-SnO_2_ and PiP-SnO_2_ thin films, respectively, where their work function and valence band maximum can be derived. (c) Steady-state photoluminescence (PL) spectral photon flux for the perovskite film with different ETLs. (d) Time-resolved confocal PL lifetime map and corresponding time-resolved PL traces for selective PiP SnO_2_-based perovskite film. (e) Transient absorption decay kinetic traces of SnO_2_/perovskite at 750 nm. The kinetic analyses with three-exponential fit are also shown by solid lines. (f) Schematic diagram of the overall photocarrier dynamics between the SnO_2_ and the perovskite.

An ideal band alignment is critical to efficiently extracting electrons from perovskite to ETL and preventing hole quenching at the ETL/perovskite interface [15,32,33]. As shown in Fig. 3a, the first principle density functional theory (DTF) calculations reveal that the removal of metastable surface OH groups reduces the conduction band minimum, facilitating charge extraction. At the same time, it increases the valence band maximum, so as to efficiently transfer electrons and block holes (Supplementary Fig. S9) [7]. Ultraviolet photoelectron spectroscopy (UPS) supports that the deeper valence band is achieved (Fig. 3b) [34]. Steady-state photoluminescence (PL) and time-resolved photoluminescence (TRPL) measurements were also carried out to probe the photocarrier dynamics between the SnO_2_ layers and the perovskite layer (Fig. 3c and SupplementaryFig. S10). The crystallinity of the deposited perovskite film on different SnO_2_ films is consistent, which is confirmed by the results of X-ray diffraction (XRD) analysis (Supplementary Fig. S11). The PiP-SnO_2_/perovskite film has a lower PL intensity and shorter carrier lifetime than the other films. Time-resolved confocal PL lifetime mapping of selectively annealed SnO_2_ by PiP-based perovskite film and the TRPL spectra of the corresponding region demonstrate the more efficient electron extraction after the PiP (Fig. 3d). The dotted box in the middle is the selectively PiP-SnO_2_/perovskite region, which exhibits a shorter PL lifetime than the original part. These results further support the reduction in charge recombination in the PiP-SnO_2_/perovskite interface. To further investigate the photocarrier dynamics at the ETL/perovskite interface, femtosecond transient absorption spectroscopy was employed, as shown in Supplementary Fig. S12, to show the time-resolved difference absorption spectra in the 10 ps–1 ns timescale of different SnO_2_/perovskite films. The amplitude of the ground-state bleaching (GSB) signal is proportional to the number of photocarriers in the excited state. This signal decays when the perovskite undergoes electron-hole recombination or charge transfer to an accepting layer [35]. It can be clearly seen that the GSB signal of the perovskite based on the PiP-SnO_2_ decays faster compared with the other two samples in the same timescale, which supports the more rapid charge transfer to the SnO_2_ ETL [36]. The decay traces of SnO_2_/perovskite have been well fitted with a three-exponential function (Fig. 3e) and the relevant decay time constants are collected in the inserted table. As shown in Fig. 3f, the fastest time constant τ_1_ more likely arises from the excitation of electrons [37]. The faster time constant τ_2_ is plausibly attributed to the electron transfer from the photoexcited perovskite to the SnO_2_ conduction band, while the slowest time constant τ_3_ is all within 400–600 ps, which can be ascribed to the charge recombination dynamics of the perovskite as reported previously [38,39]. The τ_2_ for the PiP-SnO_2_/perovskite film is much shorter, which indicates the significant enhancement of the electron-transfer rate (1/τ_2_). There is a slight increase in τ_3_, indicating that the electrons can survive for a longer time in the PiP-SnO_2_-based perovskite.

### Universality and scalability of PiP for PSCs

**Figure 4.**
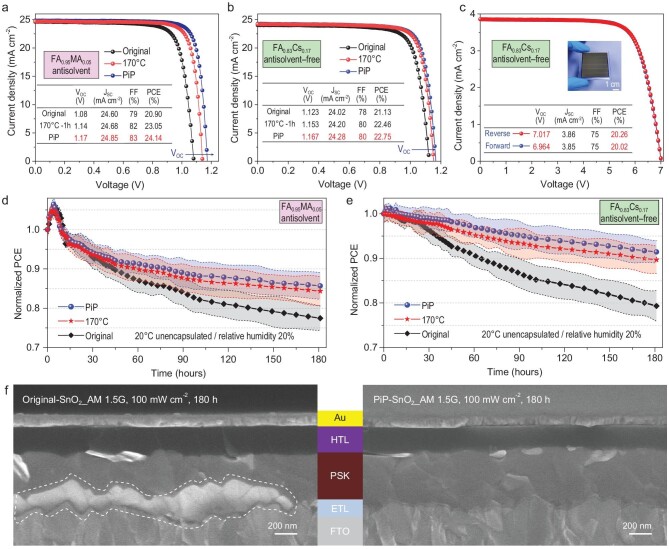
Universality and scalability of PiP for PSCs and performance comparison of PSCs. (a) *J–V* curves and photovoltaic parameters of a representative antisolvent-processed PSC that used original-SnO_2_/FA_0.95_MA_0.05_, 170°C-SnO_2_/FA_0.95_MA_0.05_ and PiP-SnO_2_/FA_0.95_MA_0.05_. (b) *J–V* curves and photovoltaic parameters of a representative antisolvent-free-processed PSC that used original-SnO_2_/FA_0.83_Cs_0.17_, 170°C-SnO_2_/FA_0.83_Cs_0.17_ and PiP-SnO_2_/FA_0.83_Cs_0.17_. (c) *J–V* curves of the PSM recorded in the lab using a mask with an aperture area of 12 cm^2^. Average PCE evolution of the unencapsulated (d) FA_0.95_MA_0.05_ and (e) FA_0.83_Cs_0.17_ devices measured over a 180-h stability test under light soaking with AM1.5G-simulated illumination (RH ∼ 20 ± 5%). The shaded regions represent the variation range of the PCE obtained from six cells. (f) Cross-sectional SEM images of original-SnO_2_/FA_0.83_Cs_0.17_/i-BABr/spiro/Au and PiP-SnO_2_/FA_0.83_Cs_0.17_/i-BABr/spiro/Au aged under continuous AM1.5G-simulated illumination for 180 hours. The degraded area of the perovskite film at the SnO_2_/perovskite interface is marked by the dashed line.

To evaluate the effect of the PiP of the SnO_2_ thin film on cell performance, we fabricated antisolvent-processed PSCs that have the layered architecture FTO/SnO_2_/(FAPbI_3_)_0.95_(MAPbBr_3_)_0.05_ (FA_0.95_MA_0.05_)/spiro-OMeTAD/Au, where MA is methylammonium and FA refers to formamidinium. The typical current density–voltage (*J*–*V*) curves are shown in Fig. 4a and Supplementary Figs S13 and S14a–c. It is worth noting that the hysteresis is suppressed after the PiP. The PiP-SnO_2_-based PSCs achieve a champion PCE of 24.14%, with an open-circuit voltage (*V_OC_*) of 1.17 V, a *J_SC_* of 24.85 mA cm^−2^ and a fill factor (FF) of 0.83. The corresponding stabilized power output (SPO) is 23.53% for the PiP-SnO_2_-based devices (Supplementary Fig. S14d) and the statistical distributions of the photovoltaic parameters are plotted in Supplementary Fig. S15. The external quantum efficiency (EQE) spectra of corresponding perovskite devices well match with the *J_SC_* measured by using the *J–V* curves (Supplementary Fig. S16).

To prove that the PiP of SnO_2_ is a universally applicable methodology, we fabricated PSCs with another type of perovskite by using antisolvent-free processing, FA_0.83_Cs_0.17_PbI_3__–__x_Cl_x_ (FA_0.83_Cs_0.17_). The representative *J–V* curves and corresponding SPOs are compared in Fig. 4b and Supplementary Fig. S17, and the statistical result is shown in Supplementary Fig. S18. The corresponding EQE spectra show an integrated *J_SC_* of 23.94 and 23.73 mA cm^−2^ for devices with and without PiP, respectively (Supplementary Fig. 19), which match well with the measured *J_SC_*. For the cell using PiP-SnO_2_, we achieve a PCE of 22.75% with negligible hysteresis. The FF increases from 78% to 80% and the *V_OC_* increases from 1.123 to 1.167 V. To demonstrate the scalability of this approach, we fabricated PSMs consisting of 6-sub cells connected in series and obtained a PCE of 20.26% on an aperture area of 12 cm^2^ with a high geometric FF (∼97.7%) by precise control of the P1–P2–P3 process (the *J–V* curves and a photograph of the PSM are shown in Fig. 4c). The details of the PSM architecture and the statistical result of the PCE are shown in Supplementary Fig. S20. An obvious increase in *V_OC_* for both FA_0.95_MA_0.05_, FA_0.83_Cs_0.17_ and FA_0.83_Cs_0.17_ PSMs is observed in Supplementary Fig. S21, which can be attributed to the reduction in the charge recombination at the SnO_2_/perovskite interface. In addition, PSCs using PiP-SnO_2_ also have improved performance compared with PSCs using SnO_2_ conventionally annealed at 170°C-SnO_2_. Furthermore, as shown in Fig. 4d and e, the long-term stability of the devices with PiP-SnO_2_ under light soaking with AM1.5G-simulated illumination at a temperature of 20 ± 5°C is greatly enhanced compared with other annealing conditions when stored under ambient air conditions with 20% ± 5% relative humidity (RH) without encapsulation over 180 hours. Upon introducing the PiP strategy to passivate the SnO_2_ layer, the bulk morphology of the SnO_2_/perovskite bilayer featuring well-defined interfaces is markedly stabilized under continuous AM1.5G-simulated illumination for 180 hours (Fig. 4f and Supplementary Fig. S22). Taken together, our results suggest that the PiP of SnO_2_ is an effective and universal approach to significantly enhance PSC performance and facilitates the scale-up of PSCs.

## CONCLUSION

In summary, in this study, we developed a novel non-equilibrium PiP technique for the low-temperature fabrication of PSCs. The ultrafast and efficient electron–electron and electron–phonon scattering ensure room-temperature annealing of SnO_2_ nanoparticle-based ETLs prepared by using CBD with an ultrafast heating rate, which efficiently passivates the surface and bulk defects of SnO_2_. By rapidly scanning a laser beam, the annealing process at room temperature can be finished in a few minutes, which normally takes hours by continuous thermal annealing at 170°C, reducing the energy cost and fabrication time. Our PiP technique has been demonstrated to be a universal and scalable approach in different types of PSCs and PSMs, and shows significantly enhanced performance in both PCE and stability (Supplementary Table S2). This study establishes a new approach towards the commercialization of efficient low-temperature manufacturing of PSCs.

## METHODS

### SnO_2_ ETL fabrication

The FTO substrate was first etched by using a femtosecond laser scanning system and then cleaned by sonicating in detergent, deionized water and ethyl alcohol for 30 min each. The CBD solution was prepared by dissolving 5 g of urea, 100 μL of mercaptoacetic acid and 5 mL of HCl (37 wt%) into 400 mL of deionized water and then adding 1.096 g of SnCl_2_⋅2H_2_O to the solution (0.012 M). The cleaned FTO substrate was immersed in the diluted CBD solution (0.002 M) and reacted at 90°C. After 3 hours, the SnO_2_ deposited FTO substrate was washed by sonicating in deionized water for 5 min. The thus prepared SnO_2_ thin film was then processed under different annealing conditions.

### The home-built PiP set-up

A femtosecond laser source (TANGOR-100 W, Amplitude) with a center wavelength of 1030/515 nm, pulse duration of ∼425 fs and oscillator frequency close to 40 MHz was integrated into a polygon laser scanning system (LSE300 STD, Next scan technology) that was equipped with a high-precision linear stage (PRO165LM, Aerotech). An attenuator (2-EWP-R-0515-M, Altechna) was placed in the optical path to adjust the laser pulse energy fluence. The polygon laser scanning speed could be up to 100 m s^−1^. To measure the exact pulse energy, a photodiode power meter was used (S120VC, Thorlabs). The in-scan resolution and the cross-scan resolution were set to 0.9 and 20 μm, respectively, so that the laser spot (45 μm) could fully cover the sample. The laser scanning speed was 36 m s^−1^ and the stage moving speed was 1.66 mm s^−1^.

### PSM laser etching

P1, P2 and P3 were all scribed by using a femtosecond laser machine (Pharos-10 W, light conversion). The FTO glass was first etched to form the module substrate with six strips (P1) with a laser power of 0.15 W, pulse duration of 10 ps, repetition rate of 10 KHz and laser scanning speed of 250 mm s^−1^. After the deposition of the spiro-OMeTAD film, the sample was re-etched to form P2 lines with a laser power of 0.3 W, pulse duration of 2 ps, repetition rate of 200 KHz and laser scanning speed of 500 mm s^−1^. Finally, an effective series of connected modules was formed by etching the Au to form P3 lines with a laser power of 5 mW, pulse duration of 260 fs, repetition rate of 1 KHz and laser scanning speed of 20 mm s^−1^. With precise control of the P1–P2–P3 process, we achieved a high geometric FF (∼97.7%).

## Supplementary Material

nwad245_Supplemental_Files

## References

[1] Chen H , Wang Y, Fan Y et al. Decoupling engineering of formamidinium–cesium perovskites for efficient photovoltaics. J Phys Chem Lett 2022; 9: 5962–9. 10.1093/nsr/nwac127 PMC9522398 36196112

[2] Chen C , Chen J, Han H et al. Perovskite solar cells based on screen-printed thin films. Nature 2022; 612: 266–71. 10.1038/s41586-022-05346-0 36352221

[3] You S , Zeng H, Liu Y et al. Radical polymeric p-doping and grain modulation for stable, efficient perovskite solar modules. Science 2023; 379: 288–94. 10.1126/science.add8786 36656941

[4] Yang T , Ma C, Cai W et al. Amidino-based Dion-Jacobson 2D perovskite for efficient and stable 2D/3D heterostructure perovskite solar cells. Joule 2023; 7: 574–86. 10.1016/j.joule.2023.02.003

[5] Li D , Shi J, Xu Y et al. Inorganic-organic halide perovskites for new photovoltaic technology. Natl Sci Rev 2018; 5: 559–76. 10.1093/nsr/nwx100

[6] Luo L , Zeng H, Wang Z et al. Stabilization of 3D/2D perovskite heterostructures via inhibition of ion diffusion by cross-linked polymers for solar cells with improved performance. Nat Energy 2023; 8: 294–303. 10.1038/s41560-023-01205-y

[7] Yoo JJ , Seo G, Chua MR et al. Efficient perovskite solar cells via improved carrier management. Nature 2021; 590: 587–93. 10.1038/s41586-021-03285-w 33627807

[8] Yu Z , Yang Z, Ni Z et al. Simplified interconnection structure based on C_60_/SnO_2-x_ for all-perovskite tandem solar cells. Nat Energy 2020; 5: 657–65. 10.1038/s41560-020-0657-y

[9] Kim M , Jeong J, Lu H et al. Conformal quantum dot-SnO_2_ layers as electron transporters for efficient perovskite solar cells. Science 2022; 375: 302–6. 10.1126/science.abh1885 35050659

[10] Bu T , Ono LK, Li J et al. Modulating crystal growth of formamidinium-caesium perovskites for over 200 cm^2^ photovoltaic sub-modules. Nat Energy 2022; 7: 528–36. 10.1038/s41560-022-01039-0

[11] Bu T , Li J, Li H et al. Lead halide-templated crystallization of methylamine-free perovskite for efficient photovoltaic modules. Science 2021; 372: 1327–32. 10.1126/science.abh1035 34140385

[12] Min H , Lee DY, Kim J et al. Perovskite solar cells with atomically coherent interlayers on SnO_2_ electrodes. Nature 2021; 598: 444–50. 10.1038/s41586-021-03964-8 34671136

[13] Park J , Kim J, Yun H-S et al. Controlled growth of perovskite layers with volatile alkylammonium chlorides. Nature 2023; 616: 724–30. 10.1038/s41586-023-05825-y 36796426

[14] Lee JH , Lee S, Kim T et al. Interfacial α-FAPbI_3_ phase stabilization by reducing oxygen vacancies in SnO_2−x_. Joule 2023; 7: 380–97. 10.1016/j.joule.2022.12.006

[15] Park SY , Zhu K. Advances in SnO_2_ for efficient and stable n-i-p perovskite solar cells. Adv Mater 2022; 34: 2110438. 10.1002/adma.202110438 35255529

[16] Jeong MJ , Moon CS, Lee S et al. Boosting radiation of stacked halide layer for perovskite solar cells with efficiency over 25%. Joule 2023; 7: 112–27. 10.1016/j.joule.2022.10.015

[17] Haghighi M , Ghazyani N, Mahmoodpour S et al. Low-temperature processing methods for tin oxide as electron transporting layer in scalable perovskite solar cells. Sol RRL 2023; 7: 2201080. 10.1002/solr.202201080

[18] Reddy SH , Di Giacomo F, Di Carlo A. Low-temperature-processed stable perovskite solar cells and modules: a comprehensive review. Adv Energy Mater 2022; 12: 2103534. 10.1002/aenm.202103534

[19] Huang L , Lou Y-H, Wang Z-K. Buried interface passivation: a key strategy to breakthrough the efficiency of perovskite photovoltaics. Small 2023; 19: 2302585. 10.1002/smll.202302585 37196420

[20] Han T-H , Tan S, Xue J et al. Interface and defect engineering for metal halide perovskite optoelectronic devices. Adv Mater 2019; 31: 1803515. 10.1002/adma.201803515 30761623

[21] Sundaram SK , Mazur E. Inducing and probing non-thermal transitions in semiconductors using femtosecond laser pulses. Nat Mater 2002; 1: 217–24. 10.1038/nmat767 12618781

[22] Kwon H , Baik S, Jang JE et al. Ultra-short pulsed laser annealing effects on MoS_2_ transistors with asymmetric and symmetric contacts. Electron 2019; 8: 222. 10.3390/electronics8020222

[23] Liu W , Luo J, Li S et al. The seeds and homogeneous nucleation of photoinduced nonthermal melting in semiconductors due to self-amplified local dynamic instability. Sci Adv 2022; 8: eabn4430. 10.1126/sciadv.abn4430 35857455 PMC9258811

[24] Tang E , Lin X, Gao G et al. Experimental research on temperature field distributions for optical lenses irradiated by femtosecond laser. Opt Laser Technol 2018; 106: 251–8. 10.1016/j.optlastec.2018.04.014

[25] Song C , Yang H, Liu F et al. Ultrafast femtosecond pressure modulation of structure and exciton kinetics in 2D halide perovskites for enhanced light response and stability. Nat Commun 2021; 12: 4879. 10.1038/s41467-021-25140-2 34385428 PMC8361179

[26] Chen Y , Zuo X, He Y et al. Dual passivation of perovskite and SnO_2_ for high-efficiency MAPbI_3_ perovskite solar cells. Adv Sci 2021; 8: 2001466. 10.1002/advs.202001466 PMC7927604 33717834

[27] Jung EH , Chen B and Bertens K et al Bifunctional surface engineering on SnO_2_ reduces energy loss in perovskite solar cells. ACS Energy Lett 2020; 5: 2796–801. 10.1021/acsenergylett.0c01566

[28] Huster N , Zanders D, Karle S et al. Additive-free spin coating of tin oxide thin films: synthesis, characterization and evaluation of tin β-ketoiminates as a new precursor class for solution deposition processes. Dalton Trans 2020; 49: 10755–64. 10.1039/D0DT01463J 32530011

[29] Jiang E , Ai Y, Yan J et al. Phosphate-passivated SnO_2_ electron transport layer for high-performance perovskite solar cells. ACS Appl Mater Interfaces 2019; 11: 36727–34. 10.1021/acsami.9b11817 31525907

[30] Costa IM , Colmenares YN, Pizani PS et al. Sb doping of VLS synthesized SnO_2_ nanowires probed by Raman and XPS spectroscopy. Chem Phys Lett 2018; 695: 125–30. 10.1016/j.cplett.2018.02.014

[31] Drabeski RG , Gunha JV, Novatski A et al. Raman and photoacoustic spectroscopies of SnO_2_ thin films deposited by spin coating technique. Vib Spectrosc 2020; 109: 103094. 10.1016/j.vibspec.2020.103094

[32] Qian Z , Chen L, Wang J et al. Manipulating SnO_2_ growth for efficient electron transport in perovskite solar cells. Adv Mater Interfaces 2021; 8: 2100128. 10.1002/admi.202100128

[33] Zhuang J , Mao P, Luan Y et al. Rubidium fluoride modified SnO_2_ for planar n-i-p perovskite solar cells. Adv Funct Mater 2021; 31: 2010385. 10.1002/adfm.202010385

[34] Tu B , Shao Y, Chen W et al. Novel molecular doping mechanism for n-doping of SnO_2_ via triphenylphosphine oxide and its effect on perovskite solar cells. Adv Mater 2019; 31: 1805944. 10.1002/adma.201805944 30697836

[35] Tvrdy K , Frantsuzov PA, Kamat PV. Photoinduced electron transfer from semiconductor quantum dots to metal oxide nanoparticles. Proc Natl Acad Sci USA 2011; 108: 29–34. 10.1073/pnas.1011972107 21149685 PMC3017152

[36] Zhong J , Wu W, Zhou Y et al. Room temperature fabrication of SnO_2_ electrodes enabling barrier-free electron extraction for efficient flexible perovskite photovoltaics. Adv Funct Mater 2022; 32: 2200817. 10.1002/adfm.202200817

[37] Zhang P , Zhu G, Shi Y et al. Ultrafast interfacial charge transfer of cesium lead halide perovskite films CsPbX_3_ (X = Cl, Br, I) with different halogen mixing. J Phys Chem C 2018; 122: 27148–55. 10.1021/acs.jpcc.8b07237

[38] Huang S-K , Wang Y-C, Ke W-C et al. Unravelling the origin of the photocarrier dynamics of fullerene-derivative passivation of SnO_2_ electron transporters in perovskite solar cells. J Mater Chem A 2020; 8: 23607–16. 10.1039/D0TA08752A

[39] Scheidt RA , Kerns E, Kamat PV. Interfacial charge transfer between excited CsPbBr_3_ nanocrystals and TiO_2_: charge injection versus photodegradation. J Phys Chem Lett 2018; 9: 5962–9. 10.1021/acs.jpclett.8b02690 30260227

